# Co-existence of *BRAF* and *NRAS* driver mutations in the same melanoma cells results in heterogeneity of targeted therapy resistance

**DOI:** 10.18632/oncotarget.12848

**Published:** 2016-10-24

**Authors:** Marieke I. G. Raaijmakers, Daniel S. Widmer, Apurva Narechania, Ossia Eichhoff, Sandra N. Freiberger, Judith Wenzina, Phil F. Cheng, Daniela Mihic-Probst, Rob Desalle, Reinhard Dummer, Mitchell P. Levesque

**Affiliations:** ^1^ Department of Dermatology, University of Zurich, University Hospital Zürich, Switzerland; ^2^ American Museum of Natural History, New York, New York, USA; ^3^ Department of Pathology, University of Zurich, University Hospital Zürich Switzerland; ^4^ Department of Dermatology, Skin and Endothelium Research Division, Medical University of Vienna, Austria

**Keywords:** melanoma, targeted therapy resistance, MAPK pathway, heterogeneity, mutation

## Abstract

Acquired chemotherapeutic resistance of cancer cells can result from a Darwinistic evolution process in which heterogeneity plays an important role. In order to understand the impact of genetic heterogeneity on acquired resistance and second line therapy selection in metastatic melanoma, we sequenced the exomes of 27 lesions which were collected from 3 metastatic melanoma patients treated with targeted or non-targeted inhibitors. Furthermore, we tested the impact of a second NRAS mutation in 7 BRAF inhibitor resistant early passage cell cultures on the selection of second line therapies.

We observed a rapid monophyletic evolution of melanoma subpopulations in response to targeted therapy that was not observed in non-targeted therapy. We observed the acquisition of *NRAS* mutations in the BRAF mutated patient treated with a BRAF inhibitor in 1 of 5 of his post-resistant samples. In an additional cohort of 5 *BRAF*-inhibitor treated patients we detected 7 NRAS mutations in 18 post-resistant samples. No NRAS mutations were detected in pre-resistant samples. By sequencing 65 single cell clones we prove that *NRAS* mutations co-occur with *BRAF* mutations in single cells. The double mutated cells revealed a heterogeneous response to *MEK*, *ERK, PI3K, AKT and multi RTK* - inhibitors.

We conclude that BRAF and NRAS co-mutations are not mutually exclusive. However, the sole finding of double mutated cells in a resistant tumor is not sufficient to determine follow-up therapy. In order to target the large pool of heterogeneous cells in a patient, we think combinational therapy targeting different pathways will be necessary.

## INTRODUCTION

The MAPK pathway, consisting of *RAS-RAF-MEK-ERK*, is a highly conserved signaling cascade in eukaryotic cells conserved from yeast to humans with many vital cellular functions, such as proliferation, differentiation, migration, and apoptosis [1]. About one-third of cancers have a deregulated MAPK pathway, either due to overexpression of receptor tyrosine kinases (RTKs), increased production of activating ligands, activating mutations in RTKs, *RAS* or *RAF* or to failure of pathway control mechanisms [1]. In cutaneous melanoma, deregulation of the MAPK pathway is mainly caused by a hyperactive mutation in *BRAF* (50% of cases) or *NRAS* (15% of cases), highlighting the important role of controlled MAPK signaling for melanocyte homeostasis [1, 2].

Targeting a hyperactivated MAPK pathway driven by mutated *NRAS* or *BRAF* with specific *BRAF*- and *MEK* inhibitors, increases the median overall survival from metastasized melanoma patients from 9 months with no therapy to approximately 14 months with successful inhibitor treatment [3]. Unfortunately, resistance to MAPK inhibition almost invariably develops [4]. Several resistance mechanisms have been described so far, which can roughly be divided into those that reactivate the MAPK pathway by circumventing the inhibitory effects of the MAPK inhibitors, or ones that activate alternative signaling pathways [5].

In the case of *BRAF* inhibitors, Shi et al identified reactivation of the MAPK pathway (70% of cases), mostly in the form of additional NRAS or KRAS mutation (18% and 7% of cases, respectively), CDKN2A loss (7% of cases), mutant BRAF amplification (19% of cases) or BRAF alternative splicing (13% of cases) as the most common resistance mechanisms. They also identified the PI3K-PTEN-AKT pathway as the second important resistance pathway (22% of their post-treatment samples contained mutations in PI3K-AKT regulatory genes) [5].

One of the more prevalent mechanisms is an additional mutation in *NRAS*, leading to reactivation of the MAPK pathway [6]. However, it has been published that *NRAS* and BRAF mutations are mutually exclusive in single cells due to self-induced apoptosis by sustained hyper-activation of the MAPK pathway [7, 8]. Consequently, resistant tumors of patients that contain both mutations concurrently may be comprised of several mutually-exclusive subclones with either the activating *BRAF* or *NRAS* mutations [7]. A recent paper showed that both mutations can co-occur in a small area (of approximately 10,000 cells) selected by laser microdissection [9], although this does not prove that the mutations can co-occur within single cells. Likewise, although double-mutated *NRAS/BRAF* melanoma cultures have been previously reported, these may still represent heterogeneous mixtures of singly-mutated melanoma cells [10, 11, 12], or may have arisen artificially through *in vitro* drug treatment and selection experiments [13].

Within a patient, various small populations of tumor cells (i.e., subclones) evolve during disease progression, which exhibit different genotypes and/or phenotypes ([14, 15, 16]). Due to these different tumor subclones within a patient (intra-patient heterogeneity), it is believed that diverse resistance mechanisms can co-exist within one patient [17, 5]. However, where these resistance mechanisms originate from and how they evolve under treatment remains poorly understood [6, 11].

To better characterize the evolution of intra-patient heterogeneity under different treatment regimens, we performed exome sequencing on multiple samples from 3 stage IV melanoma patients (cohort 1) who each received a different therapy (BRAF inhibitor (patient 1), MEK inhibitor (patient 3) or multi-receptor tyrosine kinase (patient 2)) but progressed quickly under treatment. We used formal phylogenetic methods on tumor DNA to model the evolution of intra-patient heterogeneity from primary tumors to each individual metastasis for the targeted and non-targeted therapies. In addition, we could detect the presence of an *NRAS* mutated subclone in 1 of 5 treatment-resistant tumors from the BRAF inhibitor resistant patient (patient 1). Single cell clone sequencing from the cell culture generated from this treatment resistant tumor revealed the co-occurrence of a BRAF and NRAS mutation in a single cell. This was confirmed in an additional group of 5 patients (patient 4, 5, 6, 7 and 8, cohort 2) from whom tumors after BRAF inhibitor treatment were collected and where cell cultures were generated from these tumors and showed secondary NRAS mutations. Sequencing of 65 clonal populations derived from 4 of these cell cultures showed the presence of both activating MAPK mutations in all but one subclone. Further *in vitro* work with these double-mutated cell cultures demonstrated sensitivity to *BRAF* inhibition, but heterogeneous responses to downstream MAPK inhibition, as well as to PI3K pathway inhibitors and the multi-receptor kinase inhibitor Pazopanib.

## RESULTS

### Tumor-type dependent, intra-patient heterogeneity

**Figure 1:**
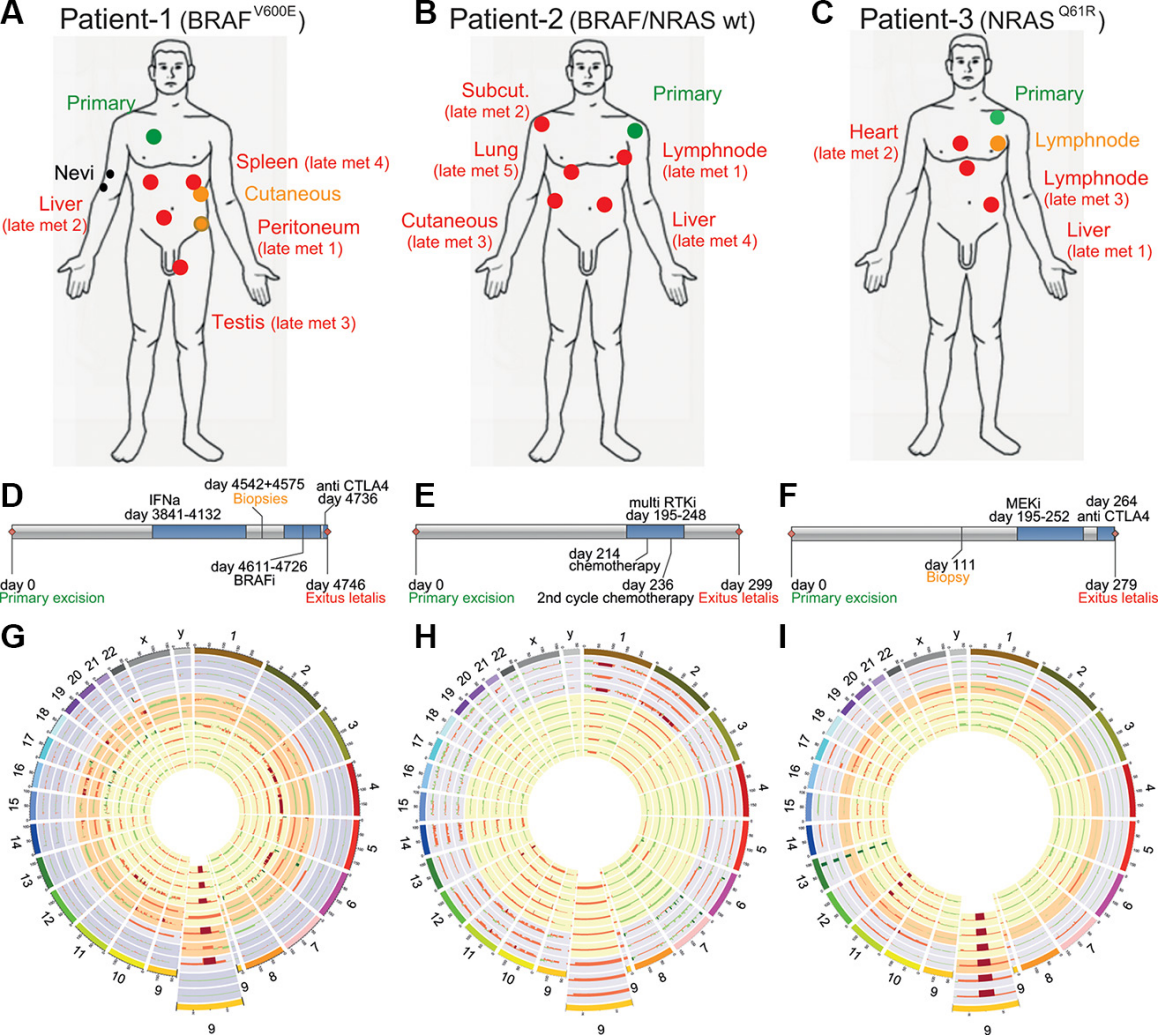
Patient cohort (A, D) Patient 1 had a *BRAFV*^600E^ mutated melanoma, samples were collected pre- and post LGX818 (encorafenib) treatment and included the primary tumor (green), two dysplastic nevi (black), two early metastases (orange) and 4 late metastases after tumor relapse (red) (**B, E**) Patient 2 had a melanoma WT for *BRAF* and *NRAS*. Samples were collected pre- and post non targeted multi RTK inhibitor (pazopanib), and included the primary tumor (green) and five late metastases (red). (**C, F**) Patient 3 had a *NRAS^Q61R^* mutated melanoma, samples were collected pre-and post MEK162 (binimetinib) treatment and included the primary tumor (green), one early metastasis (orange) and three late metastases (red). (**G**, **H**, **K**) Every ring shows the CNVs detected of one biopsy, The enlarged regions show a commonly lost region in chromosome 9 which is coding for the tumor suppressor *CDKN2A*. (G) Patient 1, rings from outside to the center represent two nevi in the two outermost circles followed by the primary tumor, the two early metastases and finally the late metastases 1 to 4. (H) Patient 2, rings from outside to the center represent primary tumor samples 1 to 3 and the late metastases 1 to 5. (**I**) Patient 3, rings from outsided to the center represent the primary tumor samples 1 and 2, one early metastases and the late metastases 1 to 3.

We sequenced whole exomes of 27 samples from three metastatic melanoma patients (cohort 1) with different mutational statuses and different treatments (Table 1 and Figure 1A–1F). For all patients we performed exome sequencing on all of their samples and confirmed their mutational status (*BRAF*^V600E^ mutated, *BRAF*^WT^*/NRAS*^WT^ or *NRAS*^Q61K^ mutated for patient 1, 2 and 3, respectively) (Table 1, Figure 1, Supplementary. Table S1). In addition to these driver mutations, we looked for other mutated onco- and tumorsuppressor genes: in patient 2 we identified a nonsynonymous germline mutation in the Melanocortin receptor *MC1R*^V92M^ and in patient 3 a nonsynonymous germline mutation in the Microphthalmia-associated transcription factor *MITF*^E318K^ (data not shown). By using EXCAVATOR and CONTRA algorithms we detected a high number of copy number variations (CNVs) in many chromosomes, with some samples exhibiting large losses throughout the genome (Figure 1G–1I).

**Table 1:** Overview table of all the patients mentioned in this manuscript

	Patient	Gender	Date of birth	Mutation identified in clinic	Treatment	Histomaterial	Mutations	Cell line	Date of original biopsy from cell line	Mutations single cells	Other mutations Cell line (MelArray)
**Cohort 1** Whole exome sequening for investigating melanoma evolution patterns under targeted vs non-targeted therapy	**Patient 1**	male	11.01.1975	BRAF V600E	LGX818 (encorafenib) Aug 2012 - Dec 2012 Drug compliance questionable	Primary Early met 1 - Early met 2 - Early met 3 - Late met 1 - Late met 2 - Late met 6 - Late met 4 - Late met 5 - Neavus 1 Neavus 2	Sanger Sequencing: NRAS WT, BRAF V600E NRAS WT, BRAF V600E NRAS WT, BRAF V600E NRAS WT, BRAF V600E NRAS WT, BRAF V600E NRAS Q61K, BRAF V600E NRAS WT, BRAF V600E NRAS WT, BRAF V600E whole exome sequencing, results see Figure 1, suppl. Table 1, Figure 2, suppl. Table 2, suppl. Table 3	MM121224	1 month after therapy skin metastasis	BRAFV600E NRAS Q61K 23 from 23 clones	ASPM C2826F GNAQ M59L HERC2 D15N KDR Y105Ter KMT2A S2819F KMT2C Q755Ter ROS1 E1291K SMARCA4 R978Q TERT promoter-146C>T
	**Patient 2**	male		BRAF WT NRAS WT	Pazopanib Oct 2012-Dec 2012	Primary (3 sites) Late met 1 Late met 2 Late met 3 Late met 4 Late met 5 Late met 6	whole exome sequencing, results see Figure 1, suppl. Table 1, Figure 2, suppl. Table 2, suppl. Table 3	-	-	-	-
	**Patient 3**	male		NRAS Q61K	MEK162 (binimetinib) Jan 2013 - Mar 2013	Primary (2 sites) Early met 1 Late met 1 Late met 2 Late met 3	whole exome sequencing, results see Figure 1, suppl. Table 1, Figure 2, suppl. Table 2, suppl. Table 3	-	-	-	-
**Cohort 2** Validating occurence of BRAF/NRAS double mutated cells	**Patient 4**	male	28.01.1959	BRAF V600E	LGX818 (encorafenib) Jul 2014 - Feb 2015	Primary - Late met 1 - Late met 2 - Late met 3 - Late met 4 -	NRAS WT, BRAF V600E NRAS Q61R, BRAF V600E NRAS WT, BRAF V600E NRAS WT, BRAF V600E NRAS Q61R, BRAF V600E	MM150423	2 months after therapy liver metastasis	BRAFV600E NRAS Q61R	ALK D885A DNMT1 L828P PLCE1 E88K
	**Patient 5**	male	16.12.1940	BRAF V600K	PLX4032 (vemurafenib) Oct 2012 - Mar 2014 LGX818 (encorafenib) in combination with MEK162 (binimetinib) Jul 2014 - Feb 2015	Late met 1 -	NRAS G12A, BRAF V600K	MM140307	1 month after BRAF inhibitor monotherapy 4 months before combination therapy skin metastasis	BRAFV600K NRAS G12A 16 from 16 clones	AKT3 R249H ASPM P223H CDC42 P87S CYP7B1 R370C EIF1AX G15D FGFR4 E560_R565del KMT2C E864G KMT2C G824D KMT2C K339N MAP2K2 F57L MAP3K1 D1084L_fs_Ter13 MAP3K9 S334C NTRK1 D537N PIK3R1 S400F PTPRK Q1364Ter ROS1 E1642K TRRAPA2383V TUSC3 Q169Ter
	**Patient 6**	female	02.03.1951	BRAF V600E	PLX4032 (vemurafenib) Aug 2013 - Aug 2014	Primary - Late met 1 - Late met 2 -	NRAS WT, BRAF V600E NRAS WT, BRAF V600E NRAS Q61K, BRAF V600E	MM140906	1 month after therapy brain metastasis	BRAFV600E NRAS Q61R	N/A
	**Patient 7**	male	26.10.1956	BRAF V600E	MEK162 (binimetinib) Nov 2011 - Feb 2012 PLX4032 (vemurafenib) Feb 2012 - Jan 2013	Early met 1 - Early met 2 - Early met 3 -	NRAS WT, BRAF V600E NRAS WT, BRAF V600E NRAS WT, BRAF V600E	M130903	8 months after PLX4032 subcutaneous metastasis	BRAFV600E NRAS Q61H 18 from 18 clones	CDKN2A R80Ter KMT2C Y987H KMT2D S3691F
	**Patient 8**	female	14.09.1974	BRAF V600E	LGX818 (encorafenib) in combination with MEK162 (binimetinib) Jul2015 - Aug 2015	Early met 1 - Early met 2 - Early met 3 - Early met 4 - Early met 5 - Early met 6 - Late met 1 - Late met 2 - Late met 3 - Late met 4 - Late met 5 - Late met 6 - Late met 7 - Late met 8 - Late met 9 - Late met 10 - Late met 11 -	NRAS WT, BRAF V600E NRAS WT, BRAF V600E NRAS WT, BRAF V600E NRAS WT, BRAF V600E NRAS WT, BRAF V600E NRAS WT, BRAF V600E NRAS Q61R, BRAF V600E NRAS WT, BRAF V600E NRAS WT, BRAF V600E NRAS WT, BRAF V600E NRAS WT, BRAF V600E NRAS Q61R, BRAF V600E NRAS WT, BRAF V600E NRAS Q61R, BRAF V600E NRAS WT, BRAF V600E NRAS WT, BRAF V600E NRAS WT, BRAF V600E	MM150849	1 month after therapy skin skull	BRAFV600E NRAS Q61R 7 from 8 clones	CDKN2A D146M_fs_Ter9 KMT2D P1170L PLCB1 E60K PTPRJ R300G SPRY4 G93R TP53 P151A TSC2 S556F
								MM150850	1 month after therapy ovary	BRAFV600E NRAS Q61K	CDC42 V36F CDKN2A D146M_fs_Ter9 KMT2C K3870R KMT2C G892R KMT2C T820I KMT2D P1170L MAP3K5 T989D_fs_Ter35 PIKFYVE Q663R_fs_Ter33 PLCB1 E60K ROS1 K832R_fs_Ter2 SPRY4 G93R TP53 P151A TSC2 S556F

Cohort 1 consists of three patients on whose immunohistochemistry material we performed whole exome sequencing in order to obtain insight in the evolution of melanoma progression under targeted and non-targeted therapy. The finding of a double mutated BRAF and NRAS cell culture was validated in cell cultures from cohort 2. Cohort 2 consists of 5 patients cell cultures showed mutations in both NRAS and BRAF. Sanger sequencing of all of the immunohistochemistry blocks from those patients could identify the double mutation only in the post-treatment samples.

### Whole-exome phylogenetic analysis identifies monophyletic evolution of therapeutic resistance

**Figure 2:**
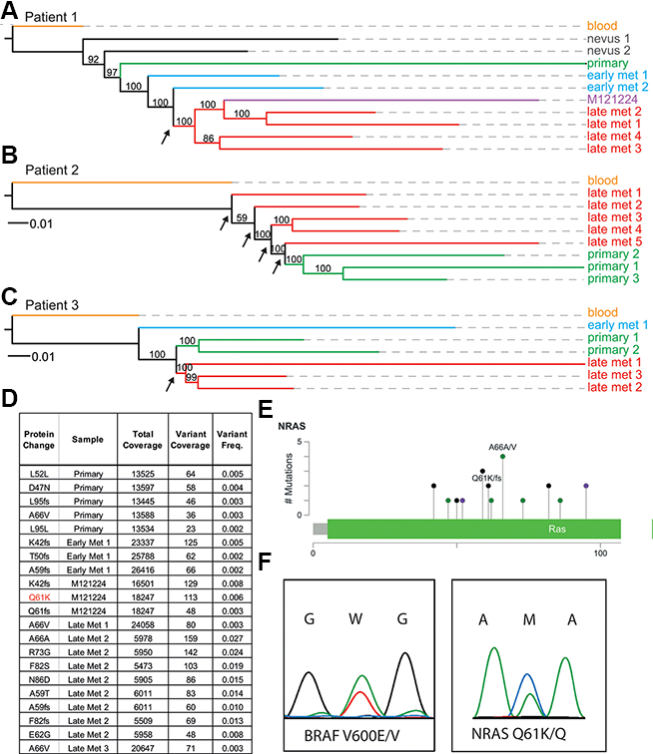
Whole-exome phylogenetic trees of patient biopsies Branch-lengths represent relative distances based on SNVs and indels, and the branches are colored according to biopsy type. Maximum likelihood phylogenetic trees are rooted by the blood sample for patient 1 (**A**), patient 2 (**B**), and patient 3 (**C**). Node supports are given as bootstra *p values*, with greater than 50% considered to be strong support. (**D**, **E**) deep sequencing results of the *NRAS* exon 2 locus in multiple samples from patient 1. (**F**) the double *BRAF^V600E^* and *NRAS^Q61K^* mutation is present in colonies derived from single cells.

In order to investigate the evolutionary relationship between tumor sites within individual patients, we used phylogenetic algorithms to group tumor samples based on their total single nucleotide variants (SNVs) and copy number variations (CNVs) (Figure 2, Supplementary Table S2). As opposed to the phylogenetic tree of patient 2 biopsies, the evolutionary trees from patient 1 and 3 showed monophyletic clades for the post-resistance samples (i.e. late metastases), meaning that these originated from only one node (Figure 2). However, no known and shared mechanism of resistance to *BRAF*-inhibitor or *MEK*-inhibitor treatment could be identified (Supplementary Table S2). Confidence is shown by bootstrap supports (arrow) which reflects the percentage of bootstrap trees also placing the clade at the endpoints of that branch. The tree of patient 2 (*BRAF*^WT^ and *NRAS*^WT^, patient received non-targeted therapy) did not show this strong monophyletic clade of late tumor metastases, instead the post-resistant samples originated from multiple nodes (Figure 2, arrows). Since there might be multiple different resistance mechanisms present in one patient, we also sought to identify explanatory protein-coding changes in any of the post-treatment samples. However, no known mechanisms of resistance were identified in the exome data of any tumor in the three patients except for the *NRAS*^Q61K^ mutation that was present in a cell line (MM121224) derived from a resistant metastasis from patient 1 (Figure 2).

### Resistant subclones are present heterogeneously and at low frequencies

In order to determine the origin of this *NRAS*^Q61K^ mutation from patient 1 we performed digital PCR as well as ultra-deep sequencing on all tumor samples (data not shown, and Figure 2D). Only one cell culture (MM121224) and one biopsy where the cell culture was derived from had an activating mutation in exon 2 of the *NRAS* gene (*NRAS*^Q61K^), with a range from 5,473× coverage of the *NRAS* exon to 26,416× coverage by next generation sequencing (Figure 2D, and Supplementary Table S3). No other sample from this patient had this activating mutation, suggesting other resistance mechanisms to be involved in the metastases of the same patient. Using the program deepSNV, a diverse series of other *NRAS* coding mutations in exon 2 was present significantly in all samples at low subclonal frequencies compared to the germline blood reference from the same patient (Figure 2D and 2E, Supplementary Table S3). Interestingly, the allele carrying the *NRAS*^Q61K^ mutation was only present at a frequency of about 6% in early passage cultures of the MM121224 cell line from this patient (Figure 2D–2F).

### Two activating MAPK mutations are present in single melanoma cells

To determine the frequency of double mutations in early passage cultures in general in a larger patient cohort, we Sanger sequenced the *NRAS* locus in all *BRAF*-mutated cell cultures generated from 2013—2015 archived in our melanoma biobank [18]. In this way, we identified an additional 8 double-mutated cell cultures, derived from 7 different patients, bringing the total to 9 out of 122 cell cultures (7.4%). Of these 9 cell cultures, 7 had an activating *BRAF* mutation at position 600 and a co-occurring activating *NRAS* mutation at position 61 or 12 (Table 1). Two of these cell cultures had a *BRAF* mutation at another position, namely one culture contained the double mutation *BRAF*^D594H^*/NRAS*^Q61R^ and one culture had the double mutation *BRAF*^L597R^*/NRAS*^Q61R^ (Data not shown). The *BRAF*^D594H^ is an inactivating mutation, and a double mutation of this sort is already described elsewhere [19]. The *BRAF*^L597R^ is a less prevalent activating mutation for which less information is available. We therefore decided to focus on the six patients (patients in cohort 2, and patient 1 from cohort 1, see Table 1) with cell cultures that had a *BRAF* mutation at position 600 and a co-occurring *NRAS* mutation at position 61 or 12.

We asked if the double-mutated cell cultures consisted of two exclusive populations of cells (one with *BRAF* and another with *NRAS* mutations), or if both mutations were present in single melanoma cells. To distinguish between these possibilities, we generated single-cell clones of 4 double mutated cell cultures (from patients 1, 5, 7 and 8) and confirmed by Sanger sequencing the presence of both *BRAF*^V600E/K^ and *NRAS*^Q61K/H/R or G12A^ mutations in 65 independently derived colonies (Table 1).

Both alleles (mutated and WT) from *BRAF* and *NRAS* were found to be expressed with Sanger sequencing of cDNA and RNA-seq (data not shown).

### Double mutations occur heterogeneously within patients after targeted therapy

In order to investigate the evolution of the *NRAS/BRAF* double mutated cancer cells within a patient, we analyzed tumor DNA for the presence of double mutations in all available histological and frozen material from patients 4, 5, 6, 7 and 8 before and after *BRAF* inhibitor treatment (Table 1). For patients 4, 5 and 6 we could confirm the presence of the additional *NRAS* mutation in the post-treatment samples from which the cell culture was derived. In patient 8, we had generated cell cultures from Late met 1 and Late met 2. In Late met 1 we could detect the *BRAF*^V600E^ *NRAS*^Q61R^ double mutation, however in Late met 2 we could not detect the additional *NRAS*^Q61K^ mutation. For patient 7, the histology block corresponding to the tumor from which the cell culture was derived was no longer available. In patient 4 and patient 8, we could also detect the *NRAS/BRAF* double mutation in additional post-treatment metastasese. Interestingly, the *NRAS* mutation could not be detected in any of the pre-treatment biopsy samples.

### Double-mutant cells have heterogeneous MAPK pathway inhibitor and alternative pathway responses

**Figure 3:**
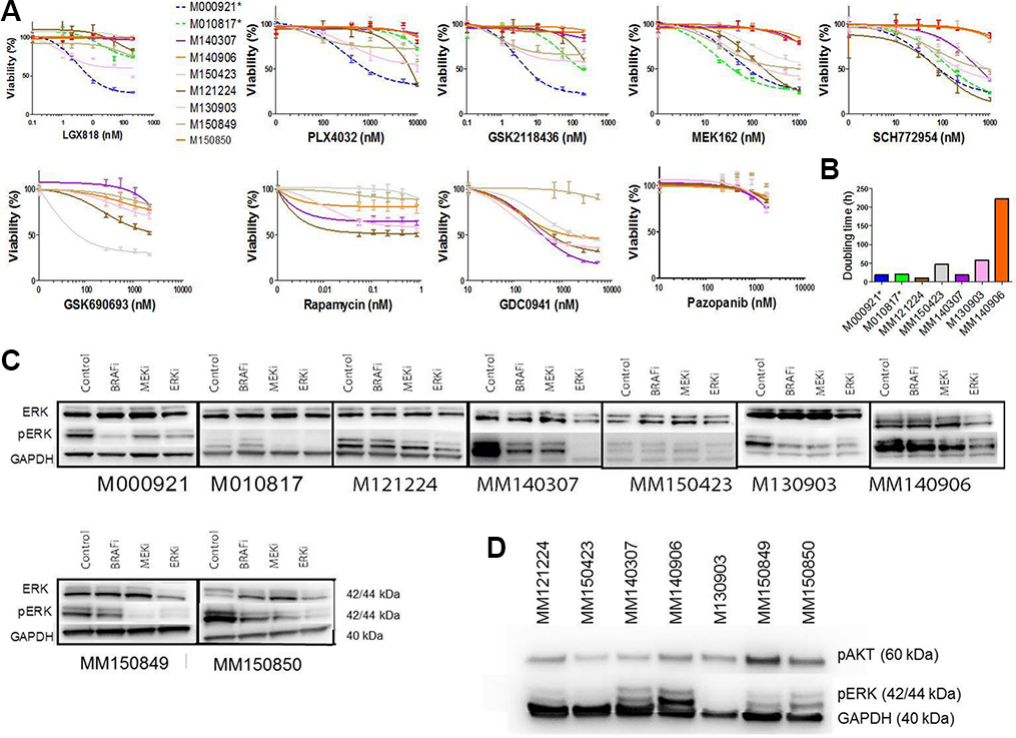
Viability and proliferation assays of double mutated cells (**A**) Viability assays of double mutated cells for different *MAPK* inhibitors and inhibitors from the PI3K-AKT pathway as well as a *multi-RTK* inhibitor. Single mutated control cell cultures are M000921 (*BRAF^V600E^*) and M010817 (*NRAS^Q61R^*), indicated in dotted lines. The double mutated cell cultures are indicated in solid lines. MM121224 (*BRAF^V600E^*, *NRAS*^Q61K^) derives from patient 1, MM140307 (*BRAF*^V600K^, NRASG12A) derives from patient 5, MM140906 (*BRAF^V600E^*, *NRAS^Q61R^*) derives from patient 6, MM150423 *(BRAF^V600E^*, *NRAS^Q61R^*) derives from patient 4, M130903 (*BRAF^V600E^*, *NRAS^Q61H^*) derives from patient 7, MM150849 (*BRAF^V600E^*, *NRAS^Q61R^*) and MM150850 (*BRAFV^600E,^ NRAS^Q61K^*) are both derived from patient 8. (**B**) Doubling time of double mutated cells under standard culturing conditions. Single mutated control cell cultures are indicated with stars. (**C**) Western blots showing *pERK* and *ERK* levels under MAPK inhibitor treatment in double mutated cell cultures. Single mutated control cell cultures are indicated with stars. (**D**) Westernblot showing pERK and pAKT levels under basic conditions (no treatment) in the different double mutated cells.

In order to gain more insight into the biology of *NRAS/BRAF* double mutated cells, we performed viability assays with (control) single and double mutated cell cultures under MAPK inhibitor treatment. Double-mutated cell cultures from all 6 identified patients were resistant to three different *BRAF* inhibitors (Figure 3A, Table 2). The response to *MEK* and *ERK* inhibitors was heterogeneous. The cell cultures MM140906 and MM150850 were resistant to MEK and ERK inhibitors, whereas other cell cultures were sensitive or partially resistant for one or for both of the inhibitors (Figure 3A, Table 2). We also tested the response for different inhibitors of the PI3K-AKT pathway, as this pathway is often involved in MAPK pathway inhibitor resistance [5], and for the multi-RTK inhibitor Pazopanib. Here the cells also showed a heterogeneous response, except for the multi-RTK inhibitor, for which all cell cultures were resistant (Figure 3A, Table 2). Therapies combining a MEK inhibitor with different PI3K pathway inhibitors worked synergistically in all cell cultures, albeit with different strengths (Table 2). As it was previously thought that *BRAF* and *NRAS* mutations are mutually exclusive due to the growth disadvantage of double mutated cells [8], we analyzed the proliferation rate of double-mutated cells *in vitro*, compared to single mutated control cell cultures (Figure 3B). Although the cell cultures MM121224 and MM140307 showed a higher proliferation rate compared to the control cell cultures, the other double-mutated cells had reduced proliferation rates.

**Table 2:** Overview table of the responses of the different double mutated cell cultures on various therapies

	MM121224		MM150423		MM140307		MM140906		M130903		MM150849		MM150850	
Drug	IC50 monotherapy	Synergism	IC50 monotherapy	Synergism	IC50 monotherapy	Synergism	IC50 monotherapy	Synergism	IC50 monotherapy	Synergism	IC50 monotherapy	Synergism	IC50 monotherapy	Synergism
PLX4032 (BRAF inhibitor)	8 uM		> 10 uM		> 10 uM		> 10 uM		> 10 uM		> 10 uM		> 10 uM	
LGX818 (BRAF inhibitor)	>200 nM		> 200 nM		> 200 nM		> 200 nM		> 200 nM		> 200 nM		> 200 nM	
GSK2118436 (BRAF inhibitor)	> 200 nM		> 200 nM		> 200 nM		> 200 nM		> 200 nM		> 200 nM		> 200 nM	
MEK162 (MEK inhibitor)	0.1 uM		1 uM		> 1 uM		> 1 uM		0.5 uM		0.5 uM		> 1 uM	
SCH772954 (ERK inhibitor)	0.05 uM		1 uM		0.5 uM		> 1 uM		0.2 uM		0.5 uM		> 1 uM	
GDC0941 (PI3K inhibitor)	500 nM		1.5 uM		500 nM		1 uM		500 nM		> 5 uM		N/A	
GSK690693 AKT inhibitor)	2 uM		100 nM		> 2 uM		> 2 uM		> 2 uM		> 2 uM		N/A	
Rapamycin (mTOR inhibitor)	0.05 nM		> 1 nM		> 1 nM		> 1 nM		0.8 nM		> 1 nM		N/A	
Pazopanib (multi RTK inhibitor)	> 2 uM		> 2 uM		> 2 uM		> 2 uM		> 2 uM		> 2 uM		N/A	
BRAFi + MEKi		+		-		-		-		+		-		N/A
ERKi + MEKi		-		+		+		++		+		-		N/A
MEKi + PI3Ki		+++		++		+		++		++		+++		N/A
MEKi + AKTi		++		++		+++		+++		++		+++		N/A
MEK + Rapamycin		+		+		+++		+++		+		++		N/A
MEK + Pazopanib		+		-		++		-		++		-		N/A

A + indicates synergism of combination treatment, with ++ and +++ being a stronger effect. A - indicates no synergy.

As a downstream read-out for *BRAF* and/or *MEK* activation, western blot analysis for total *ERK* and phosphorylated *ERK* was performed in the presence of *BRAF*, *MEK*, or *ERK* inhibition. This showed that basal *pERK* levels were mostly higher in the double mutated cells compared to the single *NRAS* mutated control cell cultures (but not in MM150423) (Figure 3C). Upon treatment with the *BRAF* inhibitor LGX818 (encorafenib), the *pERK* levels of MM121224, MM150423, MM150849 and MM140906 stayed the same compared to the untreated control, whereas the levels of MM140307, MM150850 and M130903 decreased (Figure 3C). Levels of *pERK* expression upon *MEK162 (binimetinib)* treatment decreased in all cell cultures except for MM140906. Upon *ERK* inhibition, *pERK* levels were reduced in all cell cultures.

When we compared pERK levels and pAKT levels between the different double mutated cell cultures without treatment, we found that MM140307, MM140906, MM150849 and MM150850 expressed relatively high levels of pERK, whereas M130903, MM150423 and MM121224 express relatively low levels (Figure 3D). pAKT expression is relatively constant between the different cell cultures, except MM150849, which has a relatively high expression (Figure 3D)

## DISCUSSION

Genetic or transcriptional heterogeneity in tumors is a major obstacle to obtaining durable responses to targeted therapy for metastatic melanoma. In order to better understand how individual cancer patients respond to standard therapies, we conducted multiple-sample, whole-exome sequencing from multiple time-points in 3 patients receiving different therapeutic regimens.

The sequencing results were used to infer the evolutionary relationships between the tumors within each patient, and to determine how each therapeutic regimen affected the evolution of genetic heterogeneity. Unlike previous studies that showed a branching evolution of clones subsequent to targeted therapy, we could see a strong, well-supported monophyletic evolution of metastases following both *BRAF* and *MEK* inhibitor treatment and relapse with phylogenetic analysis [5]. In contrast, patient 2, who received a multi-kinase inhibitor (i.e. pazopanib), did not have a monophyletic topology of late tumor metastases, which is suggestive of genetic drift between the late metastases. Despite the monophyletic segregation of late metastases in the patient who received the *BRAF* inhibitor, no known genetic mechanism of resistance was shared between all sequenced biopsies accounting for the inter-patient heterogeneity and subsequent treatment difficulties. In fact, the additional activating mutation in *NRAS*^Q61K^ found in patient 1 with a *BRAF* mutation background was only present in a single metastasis of patient 1 and absent in all other resistant tumor samples from that patient. This is consistent with previously published data showing heterogeneity in resistance mechanisms within individual patients [5].

We went on to check if the double mutation could also be found in the cell culture obtained from this resistant metastasis, and if these mutations occurred in the same cell. By isolating and sequencing colonies derived from 23 single-cell clones of the resistant late metastasis 6 from patient 1, we could show for the first time that both activating MAPK mutations (NRAS and BRAF) were present in a single tumor cell.

In order to confirm our finding, we identified a second cohort of BRAF inhibitor resistant patients from whom we had obtained a double mutated cell culture. In screening 121 cells from our live-cell biobank, we could identify an additional 8 cell cultures with double *BRAF/NRAS* activating mutations, bringing the total to 9 out of 122 cell cultures. By sequencing colonies derived from single-cell clones, we confirmed the presence of the double mutation in 64 out of 65 colonies. However, in our standard biobank protocol we establish cell cultures without additional treatment, also when they are derived from a therapy resistant patient. Therefore, due to selection, this could be an underrepresentation of the actual number of double mutated subclones in a typical MAPK-inhibitor resistant tumor. Sequencing of colonies derived from single cell clones of three of these double-mutated cultures confirmed that all except for one colony contained both *BRAF* and *NRAS* mutations, thus confirming the presence of both activation mutations in the same cells. Sequencing of the additional immunohistochemistry blocks from these patients only identified double mutations in the post-treatment samples, confirming the finding in patient 1 from cohort 1.

To understand the general resistance mechanisms of the double-mutated cells, we conducted viability assays with different MAPK inhibitors. The double-mutated cells grew in normal culturing conditions, notably without any MAPK inhibition, were all resistant to *BRAF*-inhibitors, but showed heterogeneity in their response to *MEK* or *ERK* inhibition, possibly because of co-existing mutations in other pathways. Combination treatment with MEK and BRAF inhibitors, as it is now clinical practice, showed synergism in MM121224 and M130903, but no synergistic or additional effect in the other cell cultures, Suggesting that simultaneous or second-line treatment with other MAPK-pathway inhibitors might still be effective in controlling progression in selected patients, but not in all. However, a MEK inhibitor combined with a PI3K, AKT or mTOR inhibitor was synergistic in all of the cell cultures, albeit with different strength. It has to be kept in mind however, as the double-mutated genotype was only present in one or two metastases from each patient, it is likely not the most important resistance mechanism in these patients and the efficacy of these second-line or combination treatments in controlling overall tumor burden is questionable. Since no common mechanism of resistance was found in any patient, it is possible that the other resistant tumors activated different pathways.

Except for 1 cell line (MM150423), all double mutated cell lines showed higher expression of *pERK* at the basal level compared to the single *NRAS* mutated cell line M010817. However, the basal level of *pERK* among the double mutated cells varied, with relatively high expression in MM140307 and MM140906 and relatively low expression in MM150423. It has been argued that *BRAF* and *NRAS* mutations are mutually exclusive due to a growth deficit of double mutated cells, because of senescence-inducing high levels of *pERK* [8]. In our experiment, MM121224 and MM140307 grew faster than the single mutated control cell lines and MM140906, MM150423 and M130903 grew slower, not supporting the view the cells with high *pERK* level grow slower. However, *in vitro* growth behavior might not represent *in vivo* growth, for instance depending on how well the cells have adapted to a 2D culture system, what growth factors are present or missing in the cell culture medium compared to the *in vivo* situation and how well the immune system can control metastasis formation.

The *pERK* levels in the cell lines under treatment of various MAPK pathway inhibitors showed some discrepancy with the proliferation assay. Most profound was the strong reduction in *pERK* upon treatment with the *BRAF* inhibitor and *MEK* inhibitor in MM140307, although the cell line was resistant to these two inhibitors. We hypothesize that this is due to the very high *pERK* levels in this cell line at baseline, in such a way that even a strong reduction compared to baseline does not suffice to block the pathway. This would also be true for MM140906 under *ERK* inhibitor treatment, although the cell line is resistant to *ERK* inhibition, levels of *pERK* show a decrease compaired to the baseline, but the levels are still high. MM150423 *pERK* levels are relatively low in *MEK* and *ERK* treated cells, although the cell line is partially resistant to these inhibitors. However, the *pERK* levels in the *MEK* and *ERK* treated cells do not differ considerably from the untreated cells.

In this study, we show that known-resistance mechanisms are present at low frequencies and heterogeneously within individual patients. Furthermore, we show that finding an additional *NRAS* mutation in a tumor sample following *BRAF* inhibitor treatment could indicate the presence of a double mutated subpopulation that is not necessarily sensitive to *MEK* inhibition or *ERK* inhibition, rendering the *MEK* inhibitor therapy in all such cases suboptimal. This study indicates that genetic analysis of one tumor biopsy does not fully define the resistance mechanism for the whole patient, which has important implications for secondary therapy strategies in case of primary resistance.

## MATERIALS AND METHODS

### Patients and sample preparation

Patients were selected after written consent from the patient, given through the university biobank program according to ethical approval numbers 647 and 800. We collected surplus material before and after therapy at autopsy. Samples were processed immediately after collection to ensure best possible DNA and RNA quality. Primary cell cultures were established as described in [18]. Notably, upon generation of cell cultures, all cultures were kept under standard conditions without additional treatment, even if they were derived from a therapy resistant patient.

DNA was isolated from paraffin embedded tissue, fresh frozen tissue, cultured cells and PMCs stored in the biobank of the institute of Dermatology of the University Hospital of Zürich. Germline DNA from PBMCs was sequenced for all patients if available as a reference [20].

DNA from paraffin blocks was isolated using the FFPE DNA isolation kit from Qiagen (QIAamp DNA FFPE Tissue Kit #56404) and optimized protocols developed by Ultan McDermott at the Sanger institute. Prior to DNA isolation, each block was evaluated by a trained dermato-histopathologist, and punches were made in tumor regions to ensure reduced contamination with stromal tissue.

For DNA isolation from non-paraffin embedded samples we followed standard DNA isolation protocols published earlier.

### Library preparation and sequencing

DNA quality was measured by an Agilent 2100 Bioanalyzer or Agilent 2200 Tapestation. One to three ug of high quality DNA was used to prepare the whole exome library using the Agilent SureSelect V4 or V5 kit. Sequencing was performed on an Illumina Hiseq 2000 machine in the Functional Genomics Center at University of Zürich. For the whole exome sequencing we sequenced 0.25 lanes per sample, paired-end, with 100 bp reads.

### Whole exome sequencing analysis

Bioinformatics analysis was conducted with a modified GATK pipeline [21–23]. Quality control was done with „FASTQC” [24]. Alignment of the FASTQ file to the reference genome “hg19” [25] Lander, Linton et al. 2001) and transformation from SAM to BAM was done with “BWA” [26]. PCR duplicates were marked by MarkDuplicates from “Picard” [22], Local realignment around indels with RealignerTargetCreator (GATK), realigning with IndelRealigner (GATK), fix mate information with FixMateInformation (Picard), base quality score recalibration with Baserecalibrator (GATK) and PrintReads (GATK). Variant calling was done with UnifiedGenotyper (GATK). For annotation of the VCF files we used Annovar [27], Samtools [28] and Bedtools [29]. For data interpretation we used Microsoft Access, Microsoft Excel, Venny [30], ConSet [31] and IGV [32, 33].

For copy number analysis we used Excavator [34] and Contra [35], results were visualized with Circos [36].

SNVs were filtered according to the following read count criteria: A base must have at least four mutant reads and at least 10 total reads, if less than 10 total reads, at least half of them must be mutated. Also all SNVs with a phred-scaled quality score of < 50 were excluded from further analysis. A SNV was called somatic if the unfiltered blood sample from the same patient did not show any mutant read for this position.

Mutant allele ratios (MAR) were calculated by dividing mutant read counts by total read counts for each called SNV. Frequencies for these ratios were calculated and trendlines were plotted in Excel with the Moving Average method (period: 3). To reduce the number of false positive SNVs we applied more strict filtering on the private SNVs. Quality threshold was raised to a phred score of 100, and the SNV needed to have at least 10 total reads. Genes that had more than 8 SNVs were excluded.

### Deep sequencing of PCR amplicons containing NRAS exon 2

DNA of 7 tumor samples (EMG P5 cell culture, M121224, 401/II, 404/II, 403, H12.684, H12.12640/1/B) were amplified with primers containing a NRAS specific sequence (see chapter sanger sequencing), adaptor sequences and a unique multiplex-identifier (MID) sequence (according to eurofins protocol). Each tumor sample analyzed is carrying therefore the adaptor sequence and a unique MID sequence. The PCR product was gel purified and 200 ng of each amplicon was sending for deep sequencing. Amplicons were subjected to Roche 454 sequencing using emulsions-PCR. Data were analyzed using DeepSNV [37].

### Sanger sequencing

After DNA amplification of *NRAS* and *BRAF* with the following primers: *BRAF* forward: 5′CTAAGAGGAAAGATG AAGTACTATG reverse: 5′CTAGTAACTCAGCAGCATCTCAG *NRAS* forward: 5′GATAGGCAGAAATGGGCTTGA reverse: 5′ATCATCCTTTCAGAGAAAATAATGC using a touchdown program going from 60 C to 55 C in 10 cycles, followed by 40 cycles at 55 C, the PCR product was diluted 100× and send to Microsynth for sequencing.

### Generation of single cell clones and single cell clone sequencing

Cells were distributed over 96-well plate, containing 1 cell per well, via FACS cell sorting or serial dilutions. Cells were grown for several weeks under standard conditions [18] until visible colonies had formed. Then, medium was removed and wells were washed with PBS. Colonies were directly lysed in the well, with 10 ul lysis buffer (2.5% 1 M Tris pH 8.0 (Ambion), 0.1% 0.5 M EDTA (Sigma-Alderich), 0.25% Tween 20 (Sigma-Alderich), 1% proteinase K (Qiagen), Aqua dest.), and incubated at 55°C for 1 hour, followed by 5 min at 95°C. Afterwards, 10 ul 25 mM MgCl2 was added and the total volume was devided over 2 PCR reactions for NRAS and BRAF Sanger sequencing.

### Cell sorting

Around 1 × 10^7^ melanoma cells were resuspended in 100 μl FACS buffer (1% FBS, 5 mM EDTA pH 8, 0.01% NaN_3_/ddH_2_O in PBS). Cells were incubated for 20 minutes at 4°C with the following photosensitive antibodies: Anti-human MCSP-FITC (1:20 dilution) (Miltenyi Biotec 130-098-794, Bergisch Gladbach Germany) and Anti-human Fibroblasts/Epithelial-PE (1:200 dilution) (ABIN319868, Aachen Germany). After washing, cells were resuspended in 200 μl FACS buffer and sorted using the Aria IIb (BD Biosciences, Franklin Lakes, New Jersey, USA).

### Phylogenetic analysis

Maximum Parsimony, Bayesian and Maximum likelihood (ML) phylogenies were constructed with the POSIX-threads version of RAxML v8.0.19 (7). To correct for among-site rate heterogeneity using the Γ distribution, we used an ascertainment bias correction and a general time reversible (GTR) substitution model. Four rate categories (ASC_GTRGAMMA model) were used to calculate the optimal tree. Node support was evaluated with 100 nonparametric bootstrap pseudoreplicates, they therefore indicate the percentage of bootstrap trees that contained a given internode branch.

Variants diagnostic for a given clade are defined as existing solely in that clade and nowhere else for that position. All leaves emanating from the node in question must share a variant and all other leaves must contain a different character for a variant to be diagnostic. Diagnostic variants can therefore also be termed an apomorphy.

### Cell viability assay

1 × 10^4^ cells were seeded and treated for 72 hours with different concentrations of either a BRAF inhibitor (PLX4032, LGX818 or GSK2118436), a MEK inhibitor (MEK162), an ERK inhibitor (SCH772984). DMSO treatment was used as a control. After 72 hours, the medium was removed and fresh RPMI1640 supplemented with 10% FCS and 8% MTT reagent (Sigma, 5 mg/ml in PBS) was added, and the cells were incubated at 37°C. After 1 hour, the RPMI1640 with MTT reagent was removed and 10% SDS (Sigma) and 95% isopropanol/ 5% Formic Acid (Sigma) (ratio 1:1) were added. After 5 min of incubation at 37°C, absorbance was measured at 595 nm (reference 620 nm) using a microplate reader.

### Synergy calculations

Combination treatments and subsequent calculation of synergy were carried out according to the method from Chou and Talalay, with the compusyn software, available at http://www.combosyn.com/index.html. We have taken the mean of the raw CI values for the different concentration combinations, in order to determine it as overall synergistic (CI value < 0.9) or overall not synergistic (CI value > 0.9).

### Proliferation assay

5 × 10^4^ cells/ml were seeded per T75 flask. After 24 hours, 72 hours, 144 hours and 240 hours the cells were counted. From the linear growth fase, the doubling time was calculated with http://www.doublingtime.com/compute.php.

### Westernblot

Total protein was collected by washing cells twice with ice cold PBS and subsequent lysis in RIPA buffer (20 mM Tris-HCl (pH 7.5), 1% Triton X-100 (Sigma), 137 mM NaCl, 10% glycerol and protease inhibitors (Roche)). Concentration of the protein was measured with the Bio-Rad Dc Protein Assay (Bio-Rad, Hercules, CA, USA) according to the manufacturer's protocol. SDS-Page was used to separate the proteins, after which they were transferred onto a nitrocellulose membrane. Membranes were probed with a rabbit anti-pERK antibody (Cell Signaling, product nr #4376S), a rabbit anti-ERK antibody (Cell Signalling, product nr#9102) and a rabbit anti-GAPDH antibody (Abcam, Cambridge, UK, product nr ab9385), followed by horseradish peroxidase-conjugated goat anti-rabbit IgG (Santa Cruz, product nr sc-2030)Bound antibodies were detected using chemiluminescence (ECL, GE Healthcare, Chalfont St. Giles, UK). Afterwards, band intensity was measured using ImageJ software (imagej.nih.gov/ij/) and pERK band intensity was corrected for corresponding GAPDH band intensity.

## SUPPLEMENTARY MATERIALS TABLES












