# Supplementary material for: Co-existence of BRAF and NRAS driver mutations in the same melanoma cells results in heterogeneity of targeted therapy resistance
Source: Oncotarget. 2016 Oct 24;7(47):77163–74. doi: 10.18632/oncotarget.12848 (PMC5363577; doi:10.18632/oncotarget.12848)
Supplement: Supplementary file 1 [file oncotarget-07-77163-s001.pdf]

Co-existence of *BRAF* and *NRAS* driver mutations in the same melanoma cells results in heterogeneity of targeted therapy resistance

Supplementary Materials

Supplementary Table S1: Somatic variants of patient 1, 2 and 3 including the cell culture MM121224 derived from late metastasis 6 of patient 1. The data in the table includes HUGO genesymbol, type of mutation, exact location of the SNV as well as reference base and mutated base. Furthermore the table comprises total read counts, read counts of reads equal to reference sequence and counts of reads which are mutated for each SNV. The listed SNVs correspond to "Somatic variants" in Supplementary Table S1. See Supplementary_Table_S1.

Supplementary Table S2: Diagnostic variants show nonsynonymous, somatic variants which are only present in resistant samples but not in any of the non-resistant samples. The data in the table include HUGO genesymbol, variant description (mut), exact location of the SNV as well as reference base and mutated base. See Supplementary_Table_S2.

Supplementary Table S3: Deep sequencing results and deepSNV analysis of multiple samples from the NRAS exon2 locus of patient 1. See Supplementary_Table_S3.

Supplementary Table S4: Raw CI values of the synergism calculations with the program from Chou and Talalay for the different drug combinations. See Supplementary_Table_S4.
